# Assessment of the relationship between bacteriological quality of dug-wells, hygiene behaviour and well characteristics in two cholera endemic localities in Douala, Cameroon

**DOI:** 10.1186/1471-2458-13-692

**Published:** 2013-07-29

**Authors:** Jane-Francis Tatah Kihla Akoachere, Lundi-Anne Omam, Thomas Njinuwo Massalla

**Affiliations:** 1 Department of Microbiology and Parasitology, Faculty of Science, University of Buea, Buea, Cameroon; 2 Laboratory for Emerging Infectious Diseases, Faculty of Science, University of Buea, Buea, Cameroon

**Keywords:** Dug-well characteristics, Water quality, Sanitation, Hygiene, Bacterial pathogens, Antibiotic susceptibility, Waterborne diseases, Extended β-lactamase producers

## Abstract

**Background:**

Access to potable water is grossly inadequate in Douala-Cameroon. The situation is worse in slum areas, compelling inhabitants to obtain water from sources of doubtful quality. This has contributed to frequent outbreaks of water-borne diseases particularly cholera, which results in severe morbidity and mortality. Shallow wells are a major source of water in these areas. We analyzed the influence of some factors on the bacteriological quality of well water in Bepanda and New Bell, cholera endemic localities in Douala to generate data that would serve as basis for strengthening of water and health policies.

**Methods:**

Questionnaires were administered to inhabitants of study sites to appraise their hygiene and sanitation practices, and level of awareness of waterborne diseases. The bacteriological quality of water was determined by investigating bacterial indicators of water quality. Relationship between well characteristics and bacteriological quality of water was determined using χ^2^ test. The Kendall *tau_b* nonparametric correlation was used to measure the strength of association between well characteristics and bacteriological parameters. Statistics were discussed at 95% confidence level. Antibiotic susceptibility of isolates was investigated by the Kirby-Bauer and broth dilution techniques. Multidrug resistant species were tested for extended β-lactamase production potential.

**Results:**

Inhabitants demonstrated adequate knowledge of waterborne diseases but employed inappropriate method (table salt) for well disinfection. Well construction and location violated guidelines. Indicator bacterial counts greatly exceeded the WHO guidelines. Variation in bacteriologic parameters between sites was not significant (P > 0.05) since well characteristics and hygiene and sanitary practices were similar. Differences in bacteriologic quality with respect to state of well, and presence of molded casing and lid, and height of casing were not significant (P > 0.05). Well distance from sanitary structure negatively correlated with bacteriological characteristics indicating it could be a major contributory factor to poor water quality. Bacteria isolated were predominantly enteric organisms. Ciprofloxacin was the most active agent. Extended β-lactamase producers were detected among *Salmonella* species, *Citrobacter fruendii* and *E. coli.*

**Conclusion:**

Poor well location, construction, and hygiene and sanitary practices were among the factors affecting water quality. There is an urgent need for education of inhabitants on effective water disinfection strategies and for regular monitoring of wells.

## Background

Water is a necessity for life. The ability to provide potable water thus affects health, ecosystems and the economy. Although many countries of the world are investing enormous resources to meet the growing demand for good quality water, the supply of potable water is still a great challenge particularly in developing countries where urbanization, industrialization and rapid population growth in the context of limited financial resources has placed a burden on water resources [1]. In these countries, lack or inadequate supply of potable water in addition to absence of basic sanitary and hygiene practices has resulted in increased morbidity and mortality from waterborne diseases particularly among children less than 5 years old [2]. In areas facing severe scarcity ground water is used as the main source of water for drinking and other purposes. Due to poverty and lack of technology to construct deep wells, majority of the population in developing countries obtain ground water from shallow wells which are easy and cheaper to construct. Unfortunately, overcrowding, poor hygiene and sanitation have subjected water in these wells to contamination with pathogenic organisms making them a potential source of health hazard.

Access to potable water in both rural and urban centers of Cameroon is a great concern [3,4]. Studies carried out in different parts of the country [5-7] show that most domestic water sources have disturbing levels of microbial pollution and this has increased the prevalence of waterborne diseases. Water related diseases have been estimated to account for two-thirds of diseases and for about 50% of deaths in Cameroon [8]. Because of this, water and sanitation has been identified by the government as one of the major pillars of the long term development goals in becoming an emerging economy by the year 2035. Therefore, studies on water quality and water conservation are of prime importance in providing data that will facilitate the government’s efforts not only in becoming an emerging economy but also to meet Target 7C of the Millennium Development Goals [9] and to improve the quality of life of Cameroonians.

In Douala, the economic capital and main port city of Cameroon, only about 650,000 persons out of a population of over 3 million [10] have access to potable water. Less than 20 million m^3^ of water is distributed annually by “Camerounaise Des Eaux” (CDE), the only company that distributes potable water in Cameroon. In parts of the city with potable water, supply is intermittent. The problem of potable water scarcity is more severe in slum areas of the city. To meet their water needs inhabitants obtain water from underground sources. Since the water table is high, hand-dug wells have served as an easier way to access ground water. Unfortunately, the quality of water from hand-dug wells is generally poor as confirmed by the regular outbreaks of waterborne diseases such as cholera which always begin from slum areas [8]. Thus frequent evaluation of well water quality and identification of potential sources of hazard is highly necessary in Douala to formulate policies and design strategies to improve water quality.

Our study was aimed at assessing some factors including well characteristics, hygiene and sanitation behavior of inhabitants, affecting the bacteriological quality of well water in Bepanda and New Bell, two localities in Douala that have always served as starting points for cholera outbreaks. The susceptibility of pathogenic or potentially pathogenic bacterial isolates to antibiotics that have been extensively used in prophylaxis or treatment of waterborne diseases in Douala was also investigated to determine suitable agents for current use especially as drug resistance has been reported [11]. Findings are expected to strengthen water and health policies and facilitate Cameroon’s achievement of health and water-related Millennium Development Goals (MDGs).

## Materials and methods

### Study sites

**Figure 1:**
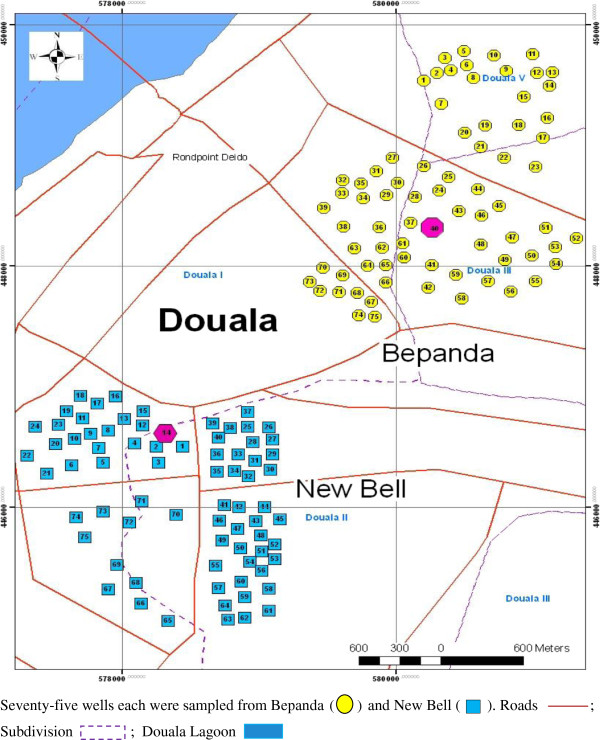
Map of study area showing sampling stations.

The study was carried out in New Bell and Bepanda (Figure 1), two densely populated unplanned neighborhoods in Douala, Littoral Region. The city of Douala has a population density of over 2000 inhabitants/km^2^[12] and housing density of 3 persons/room [13] and is located between 4°04’ at latitude North and 9°45’ at longitude East, along the coastal plain of Cameroon. Douala has an average rainfall of 2900 mm per year with an equatorial climate of two seasons: the dry season which begins in November and ends in April, with January and February being the hottest months [14], and the rainy season begins from April to October, with August and September having the highest rainfall. Temperature variations are between 26°C and 31°C. The city is poorly drained and this causes flooding during periods of heavy rains. Douala lacks a sewerage system and sewage treatment facilities hence untreated sewage is discharged indiscriminately into the environment. Although a few streams run through the study sites, dug-wells constitute the main source of water*.*

### Study design

Inhabitants’ knowledge of waterborne diseases, and their hygiene and sanitation practices was evaluated through responses to questionnaires. Dug-wells (75 each in Bepanda and New Bell) were randomly selected and their characteristics investigated. Water samples (450) were collected from these wells from February to November 2011in sterile bottles (500 ml) and their bacteriologic quality investigated. The relationship between well characteristics and bacteriological quality of samples was analyzed statistically. The antibiotic susceptibility of the isolates was also determined to ascertain effective drugs that could be used for treatment of waterborne diseases.

Ethical approval to collect data for this study was obtained from the Faculty of Health Sciences Institutional Review Board of the University of Buea (No. 2011-02-0159). Authorization to collect water samples was obtained from the District Officers of Douala Deuxieme and Douala Troisieme where study sites are located and from well owners after a thorough explanation of the purpose of the study.

### Questionnaire survey

Questionnaires (307) were administered to inhabitants of study area who use well water to obtain information on various uses of water, hygiene and sanitation practices and assess their knowledge of water borne diseases.

### Evaluation of well characteristics

The distance between perimeter of well opening and sanitary structures (pit latrines or septic tanks) was measured using a Global Positioning System (Guangzhou Making Electronic Technology Co., Ltd, China). The height of well casing above ground level was determined using a measuring tape. Other characteristics of wells such as presence of molded casing, presence of lid, and condition of well (old, fairly old or new) were determined by visual inspection.

### Sample collection

A string disinfected with 70% alcohol was tied around the neck of a sterile bottle. The bottle was lowered into the well without it touching the walls of the well. When full, the bottle was pulled out and immediately capped. Samples were labeled appropriately and placed in a box with ice packs and transported to the laboratory. Samples were processed within 24 hours of collection.

### Bacteriological analyses

#### Determination of bacterial counts

The standard plate count method was used to determine the total viable bacterial population. Coliforms were enumerated as described by Siham and Taha [15] using violet red bile lactose (VRBL) agar by pour plate technique. Plates were inoculated in duplicate. One plate was incubated at 37°C for 48 hours to isolate total coliforms and the other at 44.5°C for 48 hours to permit the growth of fecal coliforms. Colonies developed in each plate were counted.

To determine *Vibrio* counts, the method described by Shanmugam and Santhanam [16] was used. One hundred microlitres of sample was inoculated onto Thiosulphate Citrate Bile Salt Sucrose (TCBS) agar by pour plate technique. Colonies developed after incubation at 37°C for 48 hours were counted.

#### Isolation of bacteria

Samples were inoculated onto nutrient agar, MacConkey agar, mannitol salt agar, eosine methylene blue (EMB) agar and *Salmonella*-*Shigella* agar, and plates incubated at 37°C for 24 hours. Pure cultures were subjected to gram staining, oxidase test, catalase test, motility test, coagulase test (for suspected *Staphylococcu*s) and growth on Triple Sugar Iron (TSI) agar. Isolates were presumptively identified based on morphological, cultural and biochemical characteristics. The API 20E kit (BioMerieux SA, France) was used to confirm the identity of gram negative isolates.

To isolate *Vibrio cholerae* and other *Vibrio* species, samples were pre-enriched in alkaline peptone water (APW), pH 8.4, at 37°C for 24 hours. A loopful of the surface pellicle from the enriched culture was streaked on TCBS agar and incubated at 37°C for 24 hours. Yellow colonies on TCBS agar measuring 2-4 mm diameter were presumptively identified as *V. cholerae*. Green and other yellow colonies were typical of other vibrios. Isolates were characterized as described above. *V. cholerae* isolates were serotyped using *V. cholerae* O1 and O139 polyvalent antisera as described by CDC [17].

#### Antibiotic Susceptibility Testing (AST)

The disc diffusion method was used. Antibiotic discs tested were from Liofilchem Diagnostics, Italy and included tetracyclines [tetracycline (30 μg) and doxycycline (30 μg)], β-lactams [ampicillin (10 μg) and ceftriaxone (30 μg)], folic acid synthesis inhibitor [cotrimoxazole (25 μg)], fluoroquinolone [ciprofloxacin (30 μg)], protein synthesis inhibitor [chloramphenicol (30 μg)] and aminoglycoside [gentamicin (10 μg)].

MIC (minimum inhibitory concentration) was determined by the broth dilution assay [18]. MIC was determined only for antibiotics to which isolates showed sensitivity following testing by disc diffusion technique.

#### Determination of extended spectrum beta-lactamase production

Observing that all multidrug resistant isolates (resistant to 3 or more drugs) showed resistance to β-lactam antibiotics, we investigated their extended spectrum beta-lactamase (ESBL) production potential. The disc approximation method [19] was employed. An increase in the zone of inhibition due to the ceftazidime-amoxicillin-clavulanate-ceftriaxone disc synergy showed that the organism had potential for extended spectrum β-lactamase production. The diameters were compared with recommended standards, which conform to those of the Clinical Laboratory Standard Institute (CLSI) [20].

### Statistical analyses

Data was analyzed using the Statistical Package for Social Sciences (SPSS) version 17.0. Basic statistics for nominal and ordinal variables were performed using frequencies. Measures of central tendency were employed to study numeric variables and their underlying distributions. Relationship between well characteristics and water quality was assessed using Pearson’s Chi-square test. Fisher's Exact Test (FET) was used in situations where cross tabulation procedure for Chi-square produced one degree of freedom with any minimum expected count of less than 5. The Kendall *tau_b* nonparametric correlation coefficient was used to measure the strength and direction of association between scale variables. The choice of this test was because our data for well casing elevation, distance of wells from sanitary structure and bacterial counts were not normally distributed. All statistics were discussed at 95% confidence level (α, 0.05).

## Results

### Responses to questionnaires

Of the 307 questionnaires, 153 (49.8%) were administered in Bepanda and 154 (50.2%) in New Bell. Seventy-three percent (73%) of the respondents were female, majority (53.7%) of who were house wives. Most respondents had acquired at least basic education. Only one respondent (0.33%) reported using well water for drinking (Additional file 1). The rest obtained drinking water from tube wells and communal stand pipes. Uses of well water reported included cleaning (100%), cooking (73.3%) and washing of fruits and vegetables (50.4%). Inhabitants lifted well water by the bucket and rope method. One hundred and eighty two (50.9%) respondents treated their wells though not frequently, with the majority (88.5%) employing table salt as disinfectant (Additional file 1). A significant fraction of respondents (76.5%) had knowledge of well water contamination and possible sources of contamination. Use of pit latrines (93.5%) was the main method of human waste disposal. Inhabitants (64.2%) disposed domestic waste in public vats. All respondents (100%) had knowledge of waterborne diseases, with 73.0% having been victims.

### Characteristics of wells studied

Of the 150 wells sampled, only 68 (45.3%) had a lid (Table 1). The casing of nine wells (6.0%) was not raised above the ground. Only 48% of wells had casing raised ≥0.6 m above ground level. Distances between wells and sanitary structures ranged from 1 m to 17.4 m (mean distance = 7.4 m and 7.6 m respectively for Bepanda and New Bell). One hundred and eight wells (72%) were located within a distance of 10 m from a sanitary structure. Eighty-five (56.6%) wells were in a poor state, looking very old and were poorly maintained. Overall, 79.3% of the wells had a molded casing but many of these wells had cracks on the casing.

**Table 1:** Characteristics of wells studied

**Characteristic**	**Category**	**Study site**		**Total N (%)**
		**Bepanda N (%)**	**New Bell N (%)**	
**Aperture of well covered**	**Yes**	38 (25.3%)	30 (20%)	68 (45.3%)
	**No**	37 (24.7%)	45 (30%)	82 (54.7%)
**Well elevation above ground (m)**	**0.00**	3 (2.0%)	6 (4.0%)	9 (6.0%)
	**0.1-0.59**	30 (20.0%)	39 (26.0%)	69 (46.0%)
	**≥0.60**	42 (28.0%)	30 (20.0%)	72 (48.0%)
**Distance between well and sanitary structure (m)**	**0.0-10.00**	57 (38%)	51 (34%)	108 (72%)
	**10.1-15.00**	17 (11.3%)	19 (12.7%)	36(24%)
	**≥15.00**	1 (0.7%)	5(3.3%)	6(4%)
**General state of well**	**Old**	44 (29.3%	41 (27.3%)	85 (56.6%)
	**Fairly old**	26 (17.35%)	29 (19.3%)	55 (36.7%)
	**New**	5(3.3%)	5(30.3%)	10 (6.6%)
**Presence of molded casing**	**Yes**	61(81.3%)	58(77.3)	119(79.3%)
	**No**	14 (18.7%)	17(22.7%)	31(20.7%)

***N*** number of wells in Category, ***% ***percentage.

### Bacteria counts

Bacteria counts were generally high throughout the period of study. In New Bell total viable bacterial counts (TVBC) ranged from 0.0 to 4.25 × 10^4^ CFU/mL while in Bepanda, counts obtained varied from 0.0 to 1.5 × 10^4^ CFU/mL (Table 2). There was no significant difference in counts between study sites (χ^2^ = 1.931, df = 1, P = 0.165). Counts were higher in the rainy season than in the dry season. There was a significant difference in total viable bacterial counts between seasons (FET P <0.001).

**Table 2:** Bacterial population of samples with respect to site and season

**Characteristic**	**Study site**	**Season**	**Mean counts (CFU/ml)**	**SE Mean**	**StDev**	**Range (CFU/ml)**
**Heterotrophic Bacteria Counts**	**Bepanda**	**Dry**	2.28 × 10^3^	271	3068	0-1.35 × 10^4^
		**Rainy**	4.93 × 10^3^	471	4638	0-1.5 × 10^4^
	**New Bell**	**Dry**	2.82 × 10^3^	385	3554	0-1.77 × 10^4^
		**Rainy**	5.46 × 10^3^	471	5571	4.0 × 10^1^-4.25 × 10^4^
	**Total**	**Dry**	2.5 × 10^3^	224	3273	0-1.77 × 10^4^
		**Rainy**	5.252 × 10^3^	338	5206	0-4.25 × 10^4^
**Total Coliform Counts**	**Bepanda**	**Dry**	1.12 × 10^3^	139	1567	0-7.5 × 10^3^
		**Rainy**	1.84 × 10^3^	229	2256	0-1.05 × 10^4^
	**New Bell**	**Dry**	1.87 × 10^3^	253	2329	0-9.8 × 10^3^
		**Rainy**	3.20 × 10^3^	275	3259	2.0 × 10^1^-1.4 × 10^4^
	**Total**	**Dry**	1.42 × 10^3^	133	1938	0-9.8 × 10^3^
		**Rainy**	2.64 × 10^3^	192	2963	0-1.4 × 10^4^
**Fecal Coliform Counts**	**Bepanda**	**Dry**	5.89 × 10^2^	96	1087	0-7.0 × 10^3^
		**Rainy**	1.14 × 10^3^	170	1677	0-9.0 × 10^3^
	**New Bell**	**Dry**	6.25 × 10^2^	122	1124	0-6.0 × 10^3^
		**Rainy**	2.05 × 10^3^	195	2309	1.0 × 10^1^-9.0 × 10^3^
	**Total**	**Dry**	6.03 × 10^2^	75	1099	0-7.0 × 10^3^
		**Rainy**	1.68 × 10^3^	138	2119	0-9.0 × 10^3^
**Total*Vibrio*Counts**	**Bepanda**	**Dry**	4.96 × 10^2^	79	891	0-7.0 × 10^3^
		**Rainy**	8.14 × 10^2^	105	1038	0-5.0 × 10^3^
	**New Bell**	**Dry**	7.26 × 10^3^	141	1302	0-7.2 × 10^3^
		**Rainy**	1.24 × 10^3^	122	1448	2.0 × 10^1^-8.0 × 10^3^
	**Total**	**Dry**	5.88 × 10^3^	74	1077	0-7.2 × 10^3^
		**Rainy**	1.06 × 10^3^	85	1311	0-8.0 × 10^3^

***SE mean*** Standard Error of mean, ***StDev*** Standard Deviation.

Total coliform counts ranged from 0.0 to 1.05 × 10^4^ CFU/ml in Bepanda and 0.0 to 1.4 × 10^4^ CFU/ml in New Bell (Table 2). There was no significant difference in counts between sites (χ^2^ = 0.409, df =1, P = 0.522). Very high fecal coliform counts were recorded throughout the study (Table 2). In Bepanda, mean fecal coliform counts obtained were 5.89 × 10^2^ CFU/mL and 1.14 × 10^3^ CFU/mL respectively for the dry and rainy seasons. Mean counts recorded in New Bell were 6.03 × 10^2^ CFU/mL in the dry season and 2.05 × 10^3^ CFU/mL during the rainy season. Although indicator bacterial counts were generally higher in the rainy season than in the dry season, there was no significant difference in total coliform counts (FET P = 0.052) with respect to season. However, significant differences were observed in fecal coliform (FET P = 0.001) count with season.

Vibrios were detected in samples throughout the period of study with counts ranging from 0.0 to 8.0 × 10^3^ CFU/mL. Higher counts occurred in New Bell (0.0 to 8.0 × 10^3^ CFU/mL) than in Bepanda (0.0 to 7.0 × 10^3^ CFU/mL) but the difference was not significant (χ^2^ = 1.931, df = 1, P = 0.161). Counts recorded in the dry season were significantly lower (χ^2^ = 16.508, df = 1, P = 0.000) than rainy season counts.

A strong positive correlation occurred between fecal coliforms and total coliforms (+0.635) while correlations between fecal coliforms and TVBC (+0.468), and fecal coliforms and *Vibrio* counts (+0.433) though positive were weaker.

### Relationship between well characteristics and bacteriological quality of water

TVBC were higher in wells without a lid (mean = 4.73 × 10^3^ CFU/mL) than in wells with a cover (mean = 3.73 × 10^3^ CFU/mL) (Table 3). The difference was not significant (χ^2^ = 0.400, df = 1, P = 0.527). The lowest mean TVBC (3.80 × 10^3^ CFU/mL) was recorded in wells with casing elevation between 0.1 m-0.59 m. There was no significant difference in TVBC with respect to height of well casing (χ^2^ = 0.868, df = 2, P = 0.648) (Table 3). A negative correlation (−0.003) was observed between well elevation and TVBC. Total viable bacterial counts decreased with increase in distance from sanitary structure (Table 3). The lowest mean count of 2.64 × 10^3^ CFU/mL occurred in wells located at a distance greater than 15 m from sanitary structure. However, there was no significant difference (χ^2^ =0.750, df = 2, P = 0.687) in counts with respect to distance from a sanitary structure. The correlation between well distance from sanitary structure and TVBC was negative (−0.119). Highest mean TVBC occurred in old wells (4.27 × 10^3^ CFU/mL) (Table 3). There was no significant difference in counts with respect to the state of wells (χ^2^ = 3.486, df = 2, P = 0.175). Wells without a molded casing had higher TVBC (mean = 4.08 × 10^3^ CFU/mL) compared to wells with molded casing (mean = 3.932 × 10^3^ CFU/mL) (Table 3). The difference was not significant (χ^2^ =0.697, df = 1, P = 0.697).

**Table 3:** Relationship between well characteristics and heterotrophic bacterial load of samples

**Well characteristic**	**Category**	**Mean counts (CFU/ml)**	**SE Mean**	**StDev**	**Range (CFU/ml)**	**Correlation coefficient (P value)**
**Condition of well aperture**	**Covered**	3.73 × 10^3^	298	4253	0-2.5 × 10^4^	
	**Not covered**	4.13 × 10^3^	311	4876	0- 4.25 × 10^4^	
**Height of well casing above the ground (m)**	**0.00**	4.67 × 10^3^	933	4847	0-1.77 × 10^4^	−0.003 (0.921)
	**0.1-0.59**	3.80 × 10^3^	335	4819	0-4.25 × 10^4^	
	**≥0.60**	4.00 × 10^3^	297	4367	0-2.50 × 10^4^	
**Distance between well and sanitary structure (m)**	**1.0-10.00**	4.13 × 10^3^	266	4784	0- 4.25 × 10^4^	−0.119 (0.000)
	**10.1-15.00**	3.61 × 10^3^	402	4182	0-1.77 × 10^4^	
	**≥15.00**	2.64 × 10^3^	801	3399	2.6 × 10^4^ - 1.05 × 10^4^	
**State of well**	**New**	3.56 × 10^3^	808	4199	1.34 × 10^4^	
	**Fairly old**	3.38 × 10^3^	358	4161	4.0 × 10^1^-2.5 × 10^4^	
	**Old**	4.27 × 10^3^	286	4834	4.25 × 10^4^	
**Presence of molded casing**	**No**	4.08 × 10^3^	427	4052	0-4.25 × 10^4^	
	**Yes**	3.932 × 10^3^	251	4750	2.50 × 10^4^	

Well aperture (χ^2^ = 0.400, df = 1, P = 0.527). Well height (χ^2^ = 0.868, df = 2, P = 0.648).

Distance well-pit latrine (χ^2^ = 0.750, df = 2, P = 0.687). State of well (χ^2^ = 3.486, df = 2, P = 0.175). Well casing (χ^2^ = 0.697, df = 1, P = 0.697).

Total coliform counts were higher in open wells (mean = 2.14 × 10^3^ CFU/mL) than in wells with cover (mean = 1.98 × 10^3^ CFU/mL) (Table 4). The difference was not significant (χ^2^ = 0.888, df = 1, P = 0.346). Similar mean counts (1.99 × 10^3^ CFU/mL) were observed in wells with an elevation 0.1-0.59 m and those with casing not raised. However, higher mean counts (2.15 × 10^3^ CFU/ml) were obtained in wells with casing raised above 0.6 m (Table 4). The difference in total coliform counts between wells with different elevations was not significant (χ^2^ = 0.658, df = 2, P = 0.720). A weak positive correlation (+0.041) was observed between total coliform count and well elevation. Wells located within a distance of 10 m from sanitary structure had the highest mean counts (2.24 × 10^3^ CFU/mL) of total coliforms. There was no significant difference in coliform counts with respect to distances from sanitary structure (χ^2^ = 0.568, df = 2, P = 0.753). A negative correlation (−0.154) occurred between well distance and total coliform counts. Mean counts were highest in old wells (2.13 × 10^3^ CFU/mL) (Table 4). The difference in counts with respect to state of wells was not significant (χ^2^ = 3.105, df = 2, P = 0.212). The difference in total coliform counts between wells with molded casing (mean count = 2.09 × 10^3^ CFU/ml) and those without (mean count = 2.03 × 10^3^ CFU/mL) was also not significant (χ^2^ = 2.579, df = 1, P = 0.108).

**Table 4:** Relationship between well characteristics and total coliform counts of samples

**Characteristic**	**Category**	**Mean counts (CFU/ml)**	**SE Mean**	**StDev**	**Range (CFU/ml)**	**Correlation coefficient (P value)**
**Condition of well aperture**	**Covered**	1.98 × 10^3^	186	2651	0-1.1 × 10^4^	
	**Not covered**	2.14 × 10^3^	163	2561	0- 1.4 × 10^4^	
**Height of well casing above the ground (m)**	**0.00**	1. 99 × 10^3^	536	2783	2.0 × 10^1^- 1.10 × 10^4^	+0.041 (0.205)
	**0.1-0.59**	1.99 × 10^3^	184	2654	0-1.40 × 10^4^	
	**≥0.60**	2.15 × 10^3^	172	2534	0-1.05 × 10^4^	−0.154 (0.000)
**Distance between well and sanitary structure (m)**	**1.0-10.00**	2.24 × 10^3^	148	2658	0- 1.40 × 10^4^	
	**10.1-15.00**	1.59 × 10^3^	226	2352	0-1.10 × 10^4^	
	**≥15.00**	1.73 × 10^3^	642	2723	6.0 × 10^1^ – 9.0 × 10^4^	
**State of well**	**New**	2.07 × 10^3^	473	2458	2.0 × 10^1^- 8.9 × 10^3^	
	**Fairly old**	1.58 × 10^3^	189	2200	0-1.10 × 10^4^	
	**Old**	2.31 × 10^3^	164	2769	0-1.40 × 10^4^	
**Presence of molded casing**	**No**	2.03 × 10^3^	262	2483	2.0 × 10^1^- 1.40 × 10^4^	
	**Yes**	2.09 × 10^3^	140	2641	0-1.10 × 10^3^	

Well aperture (χ^2^ = 0.888, df = 1, P = 0.346). Well height (χ^2^ = 0.658, df = 2, P = 0.720). Distance well-pit latrine (χ^2^ = 0.568, df = 2, P = 0.753). State of well (χ^2^ = 3.105, df = 2, P = 0.212). Well casing (χ^2^ = 2.579, df = 1, P = 0.108).

Fecal coliform counts were significantly higher (χ^2^ = 4.202, df = 1, P = 0.040) in open wells (mean = 1.18 × 10^3^ CFU/mL) than in wells with a lid (mean = 1.15 × 10^3^ CFU/mL) (Table 5). Wells with casing not raised above ground (0.00 m) had the lowest mean count (1.01 × 10^3^ CFU/ml) (Table 5). There was no significant difference in counts with respect to well casing elevation (χ^2^ = 3.727, df = 2, P = 0.155). A weak positive correlation (+0.036) was observed between fecal coliform count and well elevation. This was not significant (P = 0.260). Counts decreased with increase in distance of well from sanitary structure. Wells located at a distance greater than 15 m had the lowest counts (mean = 7.18 × 10^2^ CFU/mL) (Table 5). However, the difference in counts with respect to distance was not significant (χ^2^ = 0.794, df = 1, P = 0.672). Fecal coliform counts negatively correlated (−0.131) with well distance from sanitary structure. There was no significant difference in fecal coliform count with respect to state of well (χ^2^ = 1.193, df = 2, P = 0.551). Higher mean counts were found in wells with molded casing (1.19 × 10^3^ CFU/ml) than those without (1.06 × 10^3^ CFU/mL) (Table 5) but the difference was not significant (χ^2^ = 0.925, df = 1, P = 0.336).

**Table 5:** Relationship between well characteristics and fecal coliform counts of samples

**Characteristic**	**Category**	**Mean counts (CFU/ml)**	**SE Mean**	**St Dev**	**Range (CFU/ml)**	**Correlation coefficient (P value)**
**Condition of well aperture**	**Covered**	1.15 × 10^3^	132	1883	0-9.0 × 10^3^	
	**Not covered**	1.18 × 10^3^	110	1722	0-9.0 × 10^3^	
**Height of well casing above the ground (m)**	**0.00**	1.01 × 10^3^	332	1726	2.0- 7.0 × 10^3^	+0.036 (0.260)
	**0.1-0.59**	1.13 × 10^3^	126	1807	0-9.0 × 10^3^	
	**≥0.60**	1.23 × 10^3^	122	1797	0-9.0 × 10^3^	
**Distance between well and sanitary structure (m)**	**1.0-10.00**	1.27 × 10^3^	103	1851	0-9.0 × 10^3^	+0.131 (0.000)
	**10.1-15.00**	9.41 × 10^2^	159	1649	0- 9.0 × 10^3^	
	**≥15.00**	7.18 × 10^2^	346	1468	0-5.5 × 10^3^	
**State of well**	**New**	1.30 × 10^3^	383	1990	0-8.0 × 10^3^	
	**Fairly old**	8.53 × 10^2^	132	1539	0-9.0 × 10^3^	
	**Old**	1.30 × 10^3^	111	1880	0-9.0 × 10^3^	
**Presence of molded casing**	**No**	1.06 × 10^3^	190	1805	0-9.0 × 10^3^	
	**Yes**	1.19 × 10^3^	95	1800	0-9.0 × 10^3^	

Well aperture (χ^2^ = 4.202, df = 1, P = 0.040). Well height: FCC (χ^2^ =3.727, df = 2, P = 0.155).

Distance well-pit latrine (χ^2^ = 0.794, df = 1, P = 0.672). State of well (χ^2^ = 1.193, df = 2, P = 0.551). Well casing (χ^2^ = 0.925, df = 1, P = 0.336).

*Vibrio* counts were higher in open wells (mean = 9.42 × 10^2^ CFU/ml) than in wells with a cover (mean = 7.19 × 10^2^. CFU/mL) (Table 6). The difference was significant (χ^2^ = 9.574, df = 1, P = 0.002). Wells with casing not raised above the ground recorded the highest *Vibrio* counts (mean = 1.0 × 10^3^. CFU/mL). The lowest counts were from wells with an elevation greater than 0.6 m (Table 6). There was no significant difference in counts with respect to height of well casing (χ^2^ = 0.767, df = 2, P = 0.682). *Vibrio* counts negatively correlated (−0.023) with well casing elevation. Similar to other bacteriologic parameters, *Vibrio* counts decreased with increase in distance from sanitary structure, with lowest counts recorded in wells located ≥15 m (Table 6). There was no significant difference in counts with respect to distance from sanitary structure (χ^2^ = 2.852, df = 2, P = 0.240). Counts negatively correlated (−0.157) with distance from sanitary structure. Higher mean counts were recorded in old wells (9.50 × 10^2^ CFU/mL) (Table 6). The difference was significant (χ^2^ = 15.435, df = 2, P < 0.001). The difference between counts in wells with molded casing and those without was not significant (χ^2^ = 0.387, df = 1, P = 0.534).

**Table 6:** **Relationship between well characteristics and*Vibrio*counts of samples**

**Characteristic**	**Category**	**Mean counts (CFU/ml)**	**SE Mean**	**StDev**	**Range (CFU/ml)**	**Correlation coefficient (P value)**
**Condition of well aperture**	**Covered**	7.19 × 10^2^	82	1171	0-8.0 × 10^3^	
	**Not covered**	9.42 × 10^2^	81	1267	0-7.6 × 10^3^	
**Height of well casing above the ground (m)**	**0.00**	1.00 × 10^3^	274	1424	0-6.60 × 10^3^	−0.023 (0.483)
	**0.1-0.59**	8.58 × 10^2^	92	1322	0-8.00 × 10^3^	
	**≥0.60**	8.04 × 10^2^	75	1107	0-7.20 × 10^3^	
**Distance between wells and sanitary structure (m)**	**1.0-10.00**	9.18 × 10^2^	70	1262	0-7.6 × 10^3^	−0.157 (0.000)
	**10.1-15.00**	6.80 × 10^2^	114	1186	0-8.0 × 10^3^	
	**≥15.00**	4.16 × 10^2^	119	506	3.0 × 10^1^- 1.6 × 10^3^	
**State of well**	**New**	6.09 × 10^2^	173	899	0-3.0 × 10^3^	
	**Fairly old**	6.69 × 10^2^	97	1125	0-7.0 × 10^3^	
	**Old**	9.50 × 10^2^	77	1295	0-8.0 × 10^3^	
**Presence of molded casing**	**No**	8.78 × 10^2^	134	1271	0-8.0 × 10^3^	
	**Yes**	8.36 × 10^2^	65	1222	0-7.20 × 10^3^	

Well aperture **(**χ^2^ = 9.574, df = 1, P = 0.002).Well height: TVC (χ^2^ = 0.767, df = 2, P = 0.682).

Distance well-pit latrine: TVC (χ^2^ = 2.852, df = 2, P = 0.240). State of well (χ^2^ = 15.435, df = 2, P = 0.000). Well casing (χ^2^ = 0.387, df = 1, P = 0.534).

### Identification of isolates

A total of 936 bacteria isolated from samples were classified into 13 species based on their cultural, morphological and biochemical characteristics. *Staphylococcus aureus* (17.8%), *Escherichia coli* (12.6%) and *Aeromonas hydrophila* (10.4%) were the most frequently isolated organisms while *Vibrio cholerae* (2.01%) was the least (Table 7). *V. cholerae* isolates belonged to the non-O1 serogroup. In New Bell, isolates most frequently detected were *S. aureus* (8.4%), *E. coli* (8.2%) and *A. hydrophila* (5.8%). Predominant isolates in Bepanda included *S. aureus* (9.5%), *Citrobacter freundii* (5.4%) and *A. hydrophila* (4.7%).

**Table 7:** Frequency of isolation of organisms from study sites

**Isolate**	**Bepanda N (%)**	**New bell N (%)**	**Total N (%)**
*S. aureus*	89 (9.5%)	78 (8.4%)	167 (17.8%)
*E. coli*	41(4.4%)	77 (8.2%)	118 (12.6%)
*A. hydrophila*	44(4.7%)	54 (5.8%)	98 (10.4%)
*C. freundii*	51(5.4%)	37 (3.9%)	88 (9.4%)
*P. aeruginosa*	42 (4.5%)	43 (4.6%)	85 (9.1%)
*E. aerogenes*	39 (4.2%)	32 (3.4%)	71 (7.6%)
*K. pneumoniae*	30 (3.2%)	38 (4.1%)	68 (7.2%)
*S. epidermidis*	31 (3.3%)	35 (3.7%)	66 (7.0%)
*V. mimicus*	22 (2.3%)	23 (2.5%)	45 (4.8%)
*Salmonella spp*	14 (1.5%)	28 (3.0%)	42 (4.5%)
*V. fluvialis*	20 (2.1%)	18 (1.9%)	38 (4.1%)
*V. vulnificus*	17 (1.8%)	17 (1.8%)	34 (3.6%)
*V. cholerae*	10 (1.1%)	9 (1.0%)	19 (2.01%)
**Total**	**450 (47.9%)**	**489 (52.1%)**	**939(100%)**

(χ^2^ = 25.266, df = 14, P = 0.032). ***N*** number of isolates, *%* percentage.

### Antibiotic sensitivity of isolates

Ten of each of the 13 bacterial species isolated (total 130 isolates) were randomly selected and their susceptibility to antibiotics tested. Ciprofloxacin (96.2%) was the most active antibiotic. Other drugs with high potency were gentamicin (88.5%), tetracycline (76.9%) and ceftriaxone (76.9%). Ampicillin (19.2%) and cotrimoxazole (34.6%) showed low susceptibilities (Table 8). Forty-four (33.8%) isolates were multidrug resistant (resistant to 3 or more antibiotics) with *Salmonella spp* showing two resistance patterns DOX^R^COT^R^TE^R^AMP^R^CEF^R^ and DOX^R^COT^R^TE^R^AMP^R^CHL^R^ (Table 9). All *Samonella spp*, *E. coli* and *Pseudomonas aeruginosa* tested were multidrug resistant and showed resistance to ampicillin and/or ceftriaxone.

**Table 8:** Antibiotic susceptibility (%) of isolates

**Isolates**	**TE**	**CHL**	**CIP**	**AMP**	**CEF**	**SXT**	**GEN**	**DXT**
*A. Hydrophila*	100	60	100	0	100	0	100	100
*C. freundii*	100	0	100	0	0	0	100	100
*E. aerogenes*	100	100	100	40	100	100	100	0
*E. coli*	100	0	50	0	0	0	100	0
*K. pneumoniae*	100	100	100	0	100	100	100	0
*P. aeruginosa*	0	100	100	0	100	0	100	0
*S. aureus*	100	100	100	100	100	100	100	100
*S. epidermidis*	100	100	100	100	100	100	100	100
*Salmonella spp*	0	50	100	0	0	0	100	0
*V. cholerae*	100	0	100	0	100	50	0	100
*V. fluvialis*	0	100	100	0	100	0	100	100
*V. mimicus*	100	100	100	0	100	0	100	100
*V. vulnificus*	100	100	100	0	100	0	100	100
**Total**	**76.9**	**70**	**96.2**	**19.2**	**76.9**	**34.6**	**88.5**	**63.1**

*TE* tetracycline, *CHL* chloramphenicol, *CIP* ciprofloxacin, *AMP* ampicillin, *CEF* ceftriaxone, *SXT* cotrimoxazole, *GEN* gentamicin, *DXT* doxycycline.

**Table 9:** Resistance patterns of isolates

**Isolate**	**Resistance pattern**	**Number of isolates with resistance pattern**	**Percentage (%) of isolates showing resistance pattern**
*P. aeruginosa*	DOX^R^COT^R^TE^R^AMP^R^	10	100
*Salmonella spp*	DOX^R^COT^R^TE^R^AMP^R^CEF^R^	5	50
	DOX^R^COT^R^TE^R^AMP^R^CEF^R^CHL^R^	5	50
*V. fluvialis*	COT^R^TE^R^AMP^R^	4	40
*C. freundii*	COT^R^AMP^R^CEF^R^CHL^R^	5	50
*E. coli*	DOX^R^COT^R^AMP^R^CEF^R^CHL^R^	10	100
*V. cholerae*	COT^R^AMP^R^GEN^R^CHL^R^	5	50
**Total**		**44**	**33.8**

*TE* tetracycline, *CHL* chloramphenicol, *CIP* ciprofloxacin, *AMP* ampicillin, *CEF* ceftriaxone, *SXT* cotrimoxazole, *GEN* gentamicin, *DXT* doxycycline.

MICs were determined for isolates that were not multidrug resistant. MIC values varied with agent and the organism tested. All isolates were sensitive to the various antibiotics with the exception of *Staphylococcus aureus* and *Staphylococcus epidermidis* which had intermediate sensitivity to tetracycline (MIC = 7.5 μg/ml for each of the organisms) and gentamicin (MIC = 5 μg/ml each) (Table 10). Lowest MIC values were obtained for ciprofloxacin (0.12 – 0.938 μg/ml), followed by gentamicin (on *Klebsiella pneumoniae* and *Enterobacter aerogenes)* (0.313 μg/ml each)). *V. mimicus, V. vulnificus*, *A. hydrophila* and *K. pneumoniae* showed low MIC to ceftriaxone. The staphylococci had the highest MIC for all drugs tested.

**Table 10:** MIC (μg/ml) of active antibiotics to isolates tested

**Isolate**	**DOX**	**COT**	**CIP**	**TE**	**AMP**	**GEN**	**CEF**	**CHL**
*S. aureus*	3.75	12.5	0.938	7.5	5	5	7.5	7.5
*S. epidermidis*	3.75	12.5	0.938	7.5	5	5	7.5	7.5
*E. aerogenes*	ND	6.25	0.47	0.47	ND	0.313	1.88	7.5
*K. pneumoniae*	ND	3.13	0.12	0.94	ND	0.313	0.94	7.5
*V. mimicus*	3.75	ND	0.23	0.47	ND	5	0.94	7.5
*V. vulnificus*	3.75	ND	0.117	0.94	ND	5	0.47	7.5
*A. hydrophila*	3.75	ND	0.47	0.94	ND	5	0.47	7.5

*ND* Not Determined, *TE* tetracycline, *CHL* chloramphenicol, *CIP* ciprofloxacin, *AMP* ampicillin, *CEF* ceftriaxone, *SXT* cotrimoxazole, *GEN* gentamicin, *DXT* doxycycline.

### Extended spectrum β-lactamase production potential of resistant isolates

Sixty (60) multi-drug resistant isolates were screened for extended spectrum β-lactamase (ESBL) production potential. Thirty (50%) of isolates demonstrated the potential for ESBL production (Table 11). All *Salmonella spp, C. fruendii* and *E. coli* exhibited the potential for extended spectrum β-lactamase production.

**Table 11:** Extended Spectrum β-Lactamase Producing Isolates

**Isolate**	**Number tested**	**Number positive (%)**	**Number negative (%)**
*P. aeruginosa*	10	0	10 (100%)
*Salmonella spp*	10	10 (100%)	0
*C. freundii*	10	10 (100%)	0
*V. cholerae*	10	0	10 (100%)
*V. fluvialis*	10	0	10 (100%)
*E. coli*	10	10 (100%)	0
**Total**	**60**	**30 (50%)**	**30 (50%)**

## Discussion

In developing countries increase in human population and industrialization has exerted an enormous pressure on the provision of safe drinking water. Provision of high quality water as well as protecting and conserving scarce water resources is therefore one of the greatest challenges currently facing national and regional governments [21]. We report here the factors affecting the bacteriological quality of well water in New Bell and Bepanda in Douala, Cameroon. This information will strengthen efforts in the provision of safe water to inhabitants of study sites.

Total viable bacterial counts (0–4.25 × 10^4^ CFU/mL), total coliform counts (0–1.4 × 10^4^ CFU/mL), fecal coliform (0–9.0 × 10^3^ CFU/mL) and *Vibrio* counts (0–8.0 × 10^3^ CFU/mL) were high throughout the period of study (Table 1). Of the 150 wells sampled, one well (in Bepanda) recorded no bacterial contamination (0 CFU/mL) throughout the study period. In New Bell, bacterial counts of 0 CFU/mL were obtained from one well only during the dry season. These wells recording 0 CFU/ml were disinfected by chlorination and used as a source of drinking water. In New Bell, chlorination was abandoned during the rainy season and bacterial contamination was detected in water. Samples from other wells were heavily contaminated and did not meet the WHO [22] standards that stated that coliforms or fecal coliform must not be detectable in any 100 mL of drinking water. TVBC exceeded the EPA [23] limit of 1.0 × 10^2^ CFU/mL. These findings are worrisome. Uses of well water reported included laundry and washing of kitchen utencils (100%), cooking (73%) and washing of fruits and vegetables (50.4%) (Additional file 1). These could predispose to waterborne infections. High counts of indicator bacteria suggest heavy pollution of water with fecal matter. Recent studies in other developing countries [24,25] have also reported high TVBC, total coliform and fecal coliforms in ground water. Significant differences (P < 0.05) in bacterial counts were observed between seasons, with higher counts recorded in the rainy season than in the dry season (Table 2). The city of Douala is poorly drained. During heavy rains, most parts of the study area are flooded and this could result in well contamination. Rainfall and flooding events have been shown to negatively impact well water quality [26] resulting in outbreaks of waterborne diseases as flood water carry microbial contaminants into unprotected water sources. Inhabitants dispose domestic waste in pits (29.3%), public vats (64.2%) and in the open (6.5%). Public vats at times are not regularly emptied when full. Under such circumstances, continuous dumping of wastes results in overflow to the surrounding. During heavy rains, these wastes could be washed into water sources contributing to pollution. A strong positive correlation occurred between fecal colifoms and total colifom counts (+0.635), but correlation between total coliforms and TVBC (+0.468), fecal coliform counts and V*ibrio* counts (+0.433) though positive was weaker. This finding suggests that these organisms could have originated from same or similar sources of contamination.

Well characteristics including construction and site management have been shown to be the most important factors responsible for well vulnerability to contamination [27]. We evaluated the relationship between well characteristics and bacteriological quality of well water. Total viable bacterial counts (Table 3), total coliform counts (Table 4), fecal coliform counts (Table 5) and *Vibrio* counts (Table 6) were higher in open wells than in wells with a lid. High bacterial counts also occurred in wells irrespective of height of casing above ground (Tables 3, 4, 5 and 6). A weak negative correlation occurred between well casing elevation and TVBC (−0.003) and also with *Vibrio* counts (−0.023). This shows that increasing well height will result in only a minor decrease in these counts. However, a weak positive correlation was observed between elevation and total coliform counts (+0.041) (Table 4) and also with fecal coliform counts (+0.036) (Table 5). This was not significant (P>0.05). Thus counts of these bacteria in well water will increase only slightly with increase in well height. Bacterial counts decreased with increase in well distance from sanitary structure (Tables 3, 4, 5 and 6). A negative correlation was seen between well distance and TVBC (−0.119), total coliform counts (−0.154), fecal coliforms (−0.131) and, *Vibrio* counts (−0.121) confirming the fact that increasing the distance between wells and sanitary structure would reduce water contamination. Although respondents demonstrated a good knowledge of waterborne diseases (100%), well water contamination and possible sources of contamination (76.5%) (Additional file 1), an indication of the success of the sensitization campaigns by the Ministry of Public Health, wells were not protected. Majority of wells were not constructed following recommended guidelines of 0.6 m well casing above ground [28] and a lateral separation distance of at least 30 m from sanitary facility [29]. Eighty-two (54.7%) wells were not covered, 52% had well casing <0.6 m above ground and 96% wells were located at a distance <15 m from sanitary structure (Additional file 1). These factors may increase the risk of contamination and account for our observations. Findings of our study show that even protected wells were still exposed to contamination. Covered wells with casing raised above 0.6 m which were poorly maintained, located near sanitary structures were subject to contamination. This may explain our finding of no significant difference in counts between covered and open wells and between wells with different casing elevations. Use of pit latrines (93%) is common in study sites. The distance between wells and sanitary structure ranged from 1 to 17.4 m. Overcrowding in study sites leaves inhabitants with limited distance between wells and sanitary structures. The close proximity of wells to sanitary infrastructure (mean distance of 7.4 m and 7.6 m in Bepanda and New Bell respectively) in addition to the presence of cracks in most casings could facilitate seepage from pit latrines contaminating well water. Wells in study sites are shallow [8] and could also be easily contaminated through seepage from latrines through the sandy, porous soil in Douala. However, to confirm this, studies focusing only on protected wells need to be carried out. Thus, poor town planning, indiscriminate well siting, poor maintenance and construction of wells may account for the poor water quality in study sites. Muruka *et al*. [30] detected an indirect association between well distance from pit latrines and fecal contaminants, with a decrease of 3.38 fecal coliforms/100 mL counts for every 1 m increase in distance. Asheesh [31] did not find any association between dug-well bacteriological quality and distance to the nearest pit latrines contradicting our findings. However, similar to our findings, Adetunji and Odetokun [24] reported a negative correlation between well distance and aerobic bacterial counts. Thus, as long as these wells and sanitary structures are in close proximity, the potential health hazard posed by highly polluted water cannot be overemphasized especially as 73% of respondents reported to have been victims of waterborne disease.

Inhabitants (100%) used the bucket and rope method to lift water from wells. This method is cheaper but could introduce contaminants into water. The use of a submersible pump is recommended but is unaffordable due to restricted financial resources of inhabitants. Although wells were disinfected, 88.5% of respondents used table salt as disinfectant and frequency of disinfection was irregular (Additional file 1). This is inappropriate and explains the heavy microbial contamination recorded in wells. Well characteristics and hygiene and sanitary practices in study sites were similar, hence there were no significant differences in bacteriological quality of water between sites.

Bacteria isolated included both pathogenic and potentially pathogenic species. Overall *S. aureus* (17.8%), *E. coli* (12.6%) and *A. hydrophila* (10.4%) predominated. There was variation in predominant isolates with site (Table 7). *S. aureus,* a normal flora of the skin, is a well recognized pathogen. It has been associated with a large number of infections including food intoxication, community acquired urinary tract infections [32], conjunctivitis, scalded skin syndrome, toxic shock, respiratory infections and skin infections. *E. coli* (12.6%) the second predominant isolate is a major public health concern as it not only indicates recent contamination with fecal matter and the possible presence of intestinal pathogens but due to the fact that certain pathogenic strains of the organism such as the enteropathogenic O157:H7 responsible for several out breaks of bloody diarrhea [33] have been detected in water. *A. hydrophila* (10.4%) is an opportunistic pathogen particularly in immunocompromised individuals. They cause non-gastrointestinal infections in humans and have been isolated from drinking water even after chlorination [34]. Its presence in our samples as one of the predominant isolates is thus a cause for concern. Other enteric organisms isolated included *Citrobacter fruendii* (9.4%), *Enterobacter aerogenes* (7.6%), *Klebsiella pneumoniae* (7.2%) and *Salmonella spp* (4.5%). Their isolation further confirms the contamination of water with fecal material and the possible presence of other waterborne enteric pathogens such as viruses and protozoa not included in our study. These enteric isolates are also of public health importance as they have been associated with several infections. *Pseudomonas aeruginosa* (9.1%) is an opportunistic pathogen responsible for a wide range of acute and chronic infections [35] when introduced into areas devoid of normal defenses. It is a major cause of bacteremia, soft tissue infections, conjunctivitis, infections of burns and wounds, cystic fibrosis, endocarditis and otitis media [36].

Four species of *Vibrio* were isolated from samples: *Vibrio mimicus* (4.8%), *V. fluvialis* (4.1%) *V. vulnificus* (3.6%) and *V. cholerae* (2.01%) (Table 7) all of which have been shown to cause infections in man and aquatic organisms. *V. mimicus* is closely related to *V. cholerae* in terms of pathogenesis of infections. Pathogenic strains cause cholera-like diarrhea in man [37] as well as infections in fish [38] and have been reported to express same virulence factors as *V. cholerae*[38]. *V. fluvialis* is an important cause of cholera-like bloody diarrhea in humans and is commonly found in areas with poor sanitation [39]. *V. vulnificus* has been shown to cause gastroenteritis and is the leading cause of reported death resulting from seafood consumption [40]. Serological typing of *V. cholerae* isolates showed that they belonged to the non-O1 serogroup. Although not responsible for epidemic cholera, the presence of the non-O1 serogroup in well water cannot be overlooked as they have been associated with diarrheal disease [41]. Certain strains have been shown to contain virulence factors present in O1 strain [42]. Furthermore, non-O1 serogroup have been reported to undergo serogroup conversion resulting in emergence of new strains with pathogenic potential [43]. Recent studies carried out in our study area [44] have reported the co-existence of O1 and non-O1 strains in well water.

It is generally believed that groundwater is relatively free of microorganisms and thus fit for consumption. However, findings from present study show that water from dug wells in New Bell and Bepanda contain high numbers of pathogenic enteric bacteria. Ndjama *et al.*[4] reported a high prevalence of waterborne diseases in other parts of Douala. Our findings together with the report of Ndjama *et al.*[4] demonstrate that waterborne diseases could be a significant health challenge in Douala. However, to determine the role of our isolates in causing diarrheal disease, their enterotoxin production potential has to be investigated. Although the Ministry of Public Health carries out sensitization campaigns on well disinfection particularly during cholera outbreaks, findings from our study show an urgent need for a regular and sustained disinfection programme in study sites.

Ciprofloxacin (96.2%) was the most effective antibiotic among those tested. Other potent antibiotics included gentamicin (88.5%), tetracycline (76.9%), ceftriaxone (76.9%), chloramphenicol (70%) and doxycycline (63.1%) (Table 8). Ciprofloxacin and gentamicin are relatively expensive drugs. In addition, gentamicin is formulated as injection. These factors discourage procurement and misuse of these drugs. Drug misuse particularly through automedication is a serious public health concern in Cameroon. Susceptibility to ampicillin (19.2%), cotrimoxazole (34.6%) and to a lesser extend doxycycline (63.1%) was low. These drugs were heavily used for treatment and prophylaxis during past cholera outbreaks in Douala [45,46] and could have resulted in selection of resistant bacteria. Considering that resistance markers have the potential of spreading across bacteria species, these organisms might have acquired resistance by various mechanisms. Forty-four (33.8%) isolates were multidrug resistant (Table 9) with twenty (20) showing resistance to five or more drugs. All (100%) *P. aeruginosa*, *Salmonella spp*, and *E. coli* tested were multi-drug resistant. Occurrence of multi-drug resistant bacteria in wells thus constitutes a health threat.

Ciprofloxacin recorded the lowest MIC values (0.117- 0.938 μg/ml) on all the isolates tested and among all the drugs (Table 10). With the exception of the staphylococci, which showed intermediate susceptibility to tetracycline and gentamicin (susceptibility break point = 8.0 μg/ml respectively), MIC determination confirmed isolates to be sensitive to antibiotics. These drugs are therefore of great value in eradication of waterborne infections in study sites. We did not use a control strain in antibiotic susceptibility testing. This constitutes a limitation to our study.

Observing that all multidrug resistant isolates were resistant to ampicillin and/or ceftriaxone, we tested isolates for extended spectrum beta-lactamase (ESBL) production potential as a possible mechanism of resistance to β-lactams. ESBL production was observed in all *Salmonella* species, *Citrobacter fruendii* and *E. coli Talble 11*. (This greatly limits:in treatment of infections caused by these pathogens. Our findings show that well water could play an important role in the dissemination of these ESBL producing organisms.

## Conclusion

Poor water quality in study area could be due to inadequate hygiene and sanitation, well disinfection and well characteristics. Our findings show that even after experiencing several devastating outbreaks of cholera, waterborne diseases will continue to be a health problem in study sites if appropriate measures are not taken to improve on water quality.

### Recommendations

There is need for enforcement of public laws on siting and construction of pit latrines, and guidelines for well construction. Distribution or subsidizing the cost of chlorine and creation of a permanent structure which ensures regular disinfection of wells and not only during disease outbreak is of utmost importance. Health and hygiene and sanitation education which should emphasize on adequate water disinfection should be carried out regularly in study area. A comprehensive study which includes the bacteriological and physico-chemical characteristics of dug-wells and tube wells needs to be carried out to provide a clear picture of ground water quality in study sites.

## Abbreviations

CDE: Camerounaise des eaux; FET: Fisher’s exact test.

## Competing interests

The authors declare that they have no competing interests.

## Authors’ contributions

JTKA as principal investigator conceived, designed and coordinated the study, interpreted data and initiated the writing of the manuscript. NBLO collected samples, isolated and characterized bacteria carried out antimicrobial susceptibility testing. MTN participated in sample collection and isolation and characterization of bacteria. All authors read and approved the final manuscript.

## Pre-publication history

The pre-publication history for this paper can be accessed here:

http://www.biomedcentral.com/1471-2458/13/692/prepub

## Supplementary Material

Additional file 1 **Responses from questionnaire.** Description of data: This is a summary of responses from questionnaire administered to inhabitants of study sites to evaluate their knowledge on waterborne diseases, and their hygiene and sanitation practices. Click here for file
